# Supplementary material for: Deep learning in finance assessing twitter sentiment impact and prediction on stocks
Source: PeerJ Comput Sci. 2024 May 10;10:e2018. doi: 10.7717/peerj-cs.2018 (PMC11157597; doi:10.7717/peerj-cs.2018)
Supplement: Supplemental Information 1 [file peerj-cs-10-2018-s001.docx]

Dear reviewer and PeerJ staff

Thank you for your letter regarding the addition of authors to our manuscript titled "Deep learning in finance assessing twitter sentiment impact and prediction on stocks." .We appreciate the opportunity to address this matter. We have carefully reviewed your requirements and would like to provide the necessary information to support the inclusion of the new author, Haoling Xie, in our manuscript.

Haoling Xie, my senior colleague in the same professional field, played a significant role in the development of this manuscript. During the experimental phase, Haoling provided invaluable guidance, including suggesting the research direction and advising on the utilization of the RoBERTa model for the task at hand. Additionally, he assisted in data analysis and interpretation, offering insights that greatly contributed to the quality of the research. I believe that Haoling Xie's significant contribution to the revision process makes it necessary for him to be listed as an author, but he feels that his participation was only advisory and does not require him to be listed as an author.

During the first revision, when it was brought to my attention by the editor that the manuscript required improvement in English language proficiency, I sought Haoling's expertise. He meticulously reviewed the entire manuscript, identifying and rectifying numerous grammatical errors and addressing technical inconsistencies. His extensive revisions significantly enhanced the clarity and coherence of the manuscript, as evidenced by the tracked changes manuscript. Furthermore, following the feedback from the reviewers, Haoling continued to provide valuable input, assisting me in addressing the reviewers' comments and further refining the manuscript to meet the journal's standards. So after the first revision, I extended the invitation once more for him to join the list of authors. He agreed to be added, and all authors consented to his inclusion. Overall, Haoling Xie has made significant contributions to the manuscript. Without his assistance, the model training and experiments in this manuscript would not have been as successful.

We appreciate your attention to this matter and thank you for your patience and understanding throughout this process. Should you require any further information or clarification, please do not hesitate to contact us. We look forward to the opportunity to address any remaining concerns and to continue working towards the publication of our manuscript in PeerJ.

Thank you once again for your time and consideration.

Sincerely,

Kaifeng Guo
